# High Maternal Serum Estradiol in First Trimester of Multiple Pregnancy Contributes to Small for Gestational Age via DNMT1-Mediated CDKN1C Upregulation

**DOI:** 10.1007/s43032-021-00735-8

**Published:** 2021-09-27

**Authors:** Xiao-Ling Hu, Shuai Shi, Ning-Ning Hou, Ye Meng, Miao Li, Ai-Xia Liu, Yong-Chao Lu, Jing-Yi Li, Jian-Zhong Sheng, Yi-Min Zhu, He-Feng Huang

**Affiliations:** 1 Department of Reproductive Endocrinology, Women’s Hospital, School of Medicine, Zhejiang University, 1 Xueshi Rd, Hangzhou, 310006 Zhejiang China; 2 Department of Pathology and Pathophysiology, School of Medicine, Zhejiang University, Hangzhou, 310058 Zhejiang China; 3 The Key Laboratory of Reproductive Genetics, Ministry of Education (Zhejiang University), Hangzhou, 310006 China

**Keywords:** Estradiol, Low birth weight, Small for gestational age, CDKN1C, Estrogen response elements

## Abstract

High maternal serum estradiol (E_2_) levels in the first trimester of pregnancy are associated with a high incidence of low birth weight (LBW) and small for gestational age (SGA). This study aimed to investigate the effect of first-trimester high maternal serum E_2_ levels on fetal growth and the underlying mechanisms in multiple pregnancies. Maternal serum E_2_ levels of women at 8 weeks of gestation were measured. The expression levels of imprinted genes and *DNMT1* were determined by RT-qPCR, and *KvDMR1* methylation in embryo tissue, placenta, and newborn cord blood samples was examined by bisulfite sequencing PCR. The effect of E_2_ on *CDKN1C* expression was investigated in HTR8 cells. The incidence of SGA was significantly higher in multiple pregnancies reduced to singleton than that in primary singleton pregnancies (11.4% vs. 2.9%) (*P* < 0.01) and multiple pregnancies reduced to twins than primary twins (38.5% vs. 27.3%) (*P* < 0.01). The maternal serum E_2_ level at 8 weeks of gestation increased with the number of fetuses and was negatively correlated with offspring birth weight. *CDKN1C* and *DNMT1* expression was significantly upregulated in embryo tissue, placenta, and cord blood from multiple pregnancies. Furthermore, there was a positive correlation between *CDKN1C* mRNA expression and *KvDMR1* methylation levels. In HTR8 cells, *DNMT1* mediated the estrogen-induced upregulation of *CDKN1C*, which might contribute to SGA. To minimize the risks of LBW and SGA, our findings suggest that abnormally high maternal serum E_2_ levels should be avoided during the first trimester of multiple pregnancies from assisted reproductive technology (ART).

**Supplementary Information:**

The online version contains supplementary material available at 10.1007/s43032-021-00735-8.

## Background

Studies from diverse human cohorts and animal models have demonstrated that low birth weight (LBW) is a great risk for developing future adult chronic diseases [1, 2]. Indeed, the maternal intrauterine environment plays a critical role in fetal development and growth. However, the mechanisms by which an adverse intrauterine environment affects fetal growth are still largely unknown.

A multiple gestation pregnancy is a common iatrogenic outcome of assisted reproductive technology (ART) mainly due to ovarian hyperstimulation. The frequency of multiple births with in vitro fertilization (IVF) ranges from 25 to 50% [3]. Multiple gestation pregnancies have an increased risk of LBW, especially when more than two fetuses are present [4–6]. It has been proposed that the reduced intrauterine growth of fetuses results from the constraints imposed by uterine size and a restricted ability of the placenta to support the nutrients required for fetuses during late gestation [5]. However, the procedure of fetal reduction in early gestation cannot eliminate all the detrimental effects of multiple gestations on the remaining fetuses. Our study showed that multiple pregnancies reduced to twins after ART treatment were still associated with a higher incidence of LBW and small for gestational age (SGA) than primary twin pregnancies. Moreover, the birth weights of singleton pregnancies from reduction were significantly lower than those of primary singleton pregnancies. Epidemiological studies of different cohorts showed that LBW was associated with adverse health outcomes, such as perinatal mortality and childhood neurologic diseases [7, 8]. Furthermore, newborns of LBW have a higher incidence of hypertension, type 2 diabetes, stroke, and coronary artery disease in later adult life [7, 9].

Although safety concerns in the first trimester of multiple pregnancies in ART are an issue, the mechanisms underlying LBW have not been well studied. The dramatic increase in estradiol (E_2_) in pregnant women may influence placental function and fetal development and play an important role in the development of numerous human diseases [10, 11]. A study in rats has demonstrated that estrogenic exposure during maternal pregnancy increases the risk of breast cancer in offspring of multiple generations [12]. Our previous study found that a high maternal E_2_ environment in the first trimester was correlated with increased risks of LBW and SGA [13]. Thus, we propose that fetuses of the first trimester of multiple pregnancies exposed to an excessive E_2_ intrauterine environment may have a higher risk of LBW and SGA.

The epigenome is most susceptible to perturbation in early development [14, 15]. The adverse effects of abnormal intrauterine exposure on fetuses may lead to epigenetic dysregulation. DNA methylation is one of the best-characterized epigenetic modifications. Abnormal DNA methylation is associated with many diseases [12, 16, 17]. DNA methyltransferase 1 (*DNMT1*) is the major *DNMT* for the maintenance of methylation and catalyzes DNA methylation by transferring a methyl group from the donor S-adenosyl methionine to the fifth carbon of cytosine [16]. Ample evidence has revealed that *DNMT1* overexpression contributes to hypermethylation associated with human disease [16, 18, 19]. In cancer cells, *DNMT1* overexpression might cause abnormal hypermethylation [20].

Genomic imprinting is an epigenetic phenomenon that results in monoallelic expression of certain genes in a parent-of-origin-dependent manner. Imprinted genes play important roles in the control of fetal growth and development [21]. *CDKN1C* is one of the best-studied imprinted genes that encodes a cyclin-dependent kinase inhibitor known to negatively regulate cellular proliferation and differentiation [22]. *KvDMR1* methylation on the maternal chromosome is proposed to activate *CDKN1C*, whereas demethylation of *KvDMR1* may cause *CDKN1C* silencing [23–25]. *CDKN1C* overexpression is one of the causes of Silver–Russell syndrome (SRS), a heterogeneous disorder characterized by severe intrauterine and postnatal growth retardation [26]. In contrast, aberrant *CDKN1C* silencing is associated with Beckwith–Wiedemann syndrome (BWS), which exhibits a loss of methylation (LOM) at the maternal *KvDMR1* imprinting control region [24, 27].

In the present study, we examined the expression levels of imprinted genes in embryo tissue from multiple and singleton gestation. We further examined the expression of imprinted *CDKN1C* in neonatal umbilical cord blood (UCB) and placenta. We also investigated changes in *CDKN1C* expression in HTR8 cells treated with different E_2_ concentrations and the underlying mechanisms by which the dramatic increase of E_2_ levels in pregnant women adversely affects fetal development and growth during the first trimester of multiple pregnancies.

## Materials and Methods

### Subjects and Tissues

This study was a retrospective study on patients that underwent ART from January 2010 to June 2014 at the Assisted Reproductive Unit of the Women’s Hospital, School of Medicine, Zhejiang University (Hangzhou, China). Written informed consent was obtained from all patients who provided samples, and the study protocol was approved by the Ethics Committee of the Women’s Hospital, School of Medicine, Zhejiang University. The inclusion criteria consisted of the selective reduction of one or more gestational sacs or embryos to singletons or twins before the 12th week of gestation. Patients with obstetric complications (e.g., gestational diabetes mellitus, hypertension, and placenta previa) or parents who consumed alcohol or smoked during pregnancy were excluded from this study. The control group included assisted reproduction patients with primary singletons or twins during the same period. Primary singletons were matched by a stratified random selection. In particular, the primary singletons chosen for the control group shared a similar maternal age, sex ratio, and gestational age to the reduced singletons. Analyses were restricted to pregnancies in which the duration of gestation was 28 weeks or longer. All pregnancies were confirmed by transvaginal ultrasonography. SGA was defined as a birth weight below the 10th percentile for the gestational age at delivery.

Multifetal pregnancy reduction (MFPR) was performed as previously described [28]. MFPR was performed early in pregnancy (about 8 weeks). Under ultrasound guidance, a 17-gauge needle was inserted through the vaginal wall into the chosen sac. Suction was applied, and all the embryonic parts were aspirated. The same technique was used for additional embryos, as needed. Maternal peripheral blood samples were obtained before MFPR at 8 weeks of gestation. UCB and placenta tissues of newborns from twins were collected at birth.

In total, 195 maternal serum samples were collected, 77 aspirated embryo tissues were collected from MFPR, and 29 embryo tissues of singleton pregnancies were collected from elective terminations of unintended normal pregnancies as controls. Mononuclear cells from UCB (UCBMNC) were separated using density gradient centrifugation and stored at −80°C, as previously described [29]. All tissue samples were collected immediately after surgical removal and were snap-frozen in liquid nitrogen.

### Measurement of Estradiol

Maternal serum E_2_ levels were measured using electrochemiluminescence (Hoffman-La Roche, Mannheim, USA). The detection limit of for E_2_ was 18.4 pmol/L, and the CV% was 2.4–4.6%.

### Cell Culture

The HTR8/SVneo (HTR8) cell line was derived from a human first-trimester placenta explant immortalized by SV40 large T antigen [30]. The cells were cultured at 37°C with a 5% CO_2_ atmosphere in phenol red-free PRMI 1640 medium (Gibco, Invitrogen, NY, USA) containing 10% fetal bovine serum (FBS), 100 IU/mL penicillin, and 100 µg/mL streptomycin. For steroid starvation, cells were cultured in phenol-free PRMI 1640 containing 5% charcoal-stripped FBS (Sigma, St. Louis, MO, USA).

### Total RNA Extraction and Real-time Quantitative RT-PCR (qRT-PCR)

Total RNA was isolated from tissue samples or cells using Trizol reagent (Takara, Dalian, China). cDNA was synthesized using the PrimeScript™ RT reagent Kit (Takara). qRT-PCR was carried out with an ABI Prism 7900HT (Applied Biosystems, Foster City, CA, USA) using SYBR® Premix Ex Taq™ (Takara). The relative gene expression was normalized to glyceraldehyde-3-phosphate dehydrogenase (*GAPDH)*, as previously described [31]. The primers used in this study are listed in Supplemental Table 1.

### Methylation-Specific PCR (MSP)

Genomic DNA was extracted from tissue samples or cells using a standard phenol-chloroform extraction method and modified with sodium bisulfite using the EpiTect®Bisulfite Kit (Qiagen, USA). DNA from peripheral blood mononuclear cells treated with SssI methyltransferase (New England Biolabs, Beverly, MA, USA) was used as a positive control for methylated alleles. The methylation-specific primers for *KvDMR1* are listed in Supplemental Table 2.

### Bisulfite Sequencing PCR

Bisulfite sequencing was performed using specific tissue samples and cells. The primers used for bisulfite sequencing of the *KvDMR1* fragment are listed in Supplemental Table 3. The amplified area contained 28 CpG sites. Amplified PCR products were purified using a gel extraction kit (Takara) and ligated into the pMD19-T plasmid using the TA-cloning system (Takara). The sequences were analyzed using 3730 DNA Analyzer polymers (Applied Biosystems, Carlsbad, CA).

### E_2_ and ICI182780 Treatment of HTR8 Cells

E_2_ (Roche, Basel, Switzerland) and the estrogen receptor inhibitor ICI182780 (Sigma, St. Louis, MO, USA) were dissolved in dimethyl sulfoxide (DMSO, Sigma). HTR8 cells were seeded in 6-well plates in 2 mL of culture medium. Cells were treated with 0, 10^-9^, 10^-8^, or 10^-7^ M E_2_ for 24 h. To investigate the effects of estrogen, cells were pretreated with 10^-5^ M ICI182780 for 4 h followed by stimulation with 10^-7^ M E_2_ for 24 h.

### Gene Silencing with DNMT1 Small Interfering RNAs (siRNAs)

For *DNMT1* gene silencing, HTR8 cells were transiently transfected with *DNMT1*-specific siRNAs (Sigma) using Lipofectamine^TM^ 2000 (Invitrogen, Carlsbad, CA), according to the manufacturer’s instructions. Scrambled siRNA was used as a control. The knockdown efficiency was confirmed by qRT-PCR and western blotting.

### Western Blotting

Western blot analysis was performed as previously described [32]. Briefly, proteins were separated by SDS-PAGE, transferred to nitrocellulose membranes, and exposed to a rabbit anti-DNMT1 antibody (1:800; Santa Cruz Biotechnology, Santa Cruz, CA) for 2 h. The blots were incubated with a horseradish peroxidase-linked antirabbit IgG (1:5000; KPL, Maryland, USA) for 1 h at room temperature, and the specific protein band was visualized using the Licor Odyssey Infrared Imaging System (Licor, Lincoln, North Carolina, USA).

### Plasmid Construction and Luciferase Reporter Assay

The human *DNMT1* promoter region (−2493 to −1 bp) was inserted into the pGL3-basic plasmid (Promega, Madison, WI, USA), resulting in the *DNMT1* promoter—firefly luciferase reporter plasmid *DNMT1*-luc. The construct was verified by sequencing. HTR8 cells were co-transfected with *DNMT1*-luc and the *Renilla* luciferase plasmid pRL-TK (Promega, Southampton, UK), which served as an internal transfection control. Transfection was performed using Fugene-HP (Roche, Basel, Switzerland), as per the manufacturer’s instructions. The transfected cells were treated with 10^-7^ M E_2_ for 24 h, and luciferase activity was measured using the Dual-Luciferase Reporter Assay System (Promega). Firefly luciferase was normalized to *Renilla* luciferase activity.

### Chromatin Immunoprecipitation (ChIP) Analysis

ChIP experiments were performed as previously described with minor modifications [33]. Briefly, cells were cross-linked with 1% formaldehyde for 10 min at 37°C, and the reaction stopped by the addition of glycine to a final concentration of 0.125 M. Lysates were sonicated on ice to shear the DNA to 200–800 bp fragments. ChIP was performed with a ChIP assay kit (Millipore, Billerica, MA, USA), according to the manufacturer’s instructions. The estrogen receptor α (ERα) antibody was obtained from Upstate (Millipore). The *DNMT1* primers used for PCR are listed in Supplemental Table 4.

### Statistical Analysis

Data are presented as the mean ± standard error (SE). The two-tailed student’s *t*-test or ANOVA was used to evaluate the statistical significance of continuous parametric data. The chi-square test was used to compare categorical data. All statistical analyses were performed using SPSS 23.0 software. Statistical significance was defined as* P* < 0.05.

## Results

### The Effect of First-Trimester Multiple Pregnancies on Birth Weight

Information for all patients who underwent reduction was available. There were 105 singleton deliveries with selective reduction (< 12 weeks), 217 twin deliveries after selective reduction, 210 primary singleton births, and 864 primary twins without spontaneous or selective reduction for analysis. The mean birth weight in patients whose fetuses were reduced to singletons or twins was significantly lower than that of their corresponding controls (3226.71 ± 513.96 g vs. 3378.19 ± 473.32 g (*P* < 0.01) and 2428.80 ± 485.64 g vs. 2495.31 ± 520.06 g (*P* < 0.05), respectively). Fetus birth weights could also be affected by the gestational age at delivery. To further elucidate this issue, we analyzed the proportion of SGA infants. The incidence of SGA in multiple pregnancies reduced to singletons (11.4%, 12/105) was significantly higher than that to primary singleton pregnancies (2.9%, 6/210) (*P* < 0.01). In addition, SGA in multiple pregnancies reduced to twins (38.5%, 167/434) was significantly higher than that with primary twin pregnancies (27.3%, 472/1728) (*P* < 0.01).

### Maternal Serum E_2_ Levels were Significantly Elevated with Increasing Number of Fetuses

**Fig. 1:**
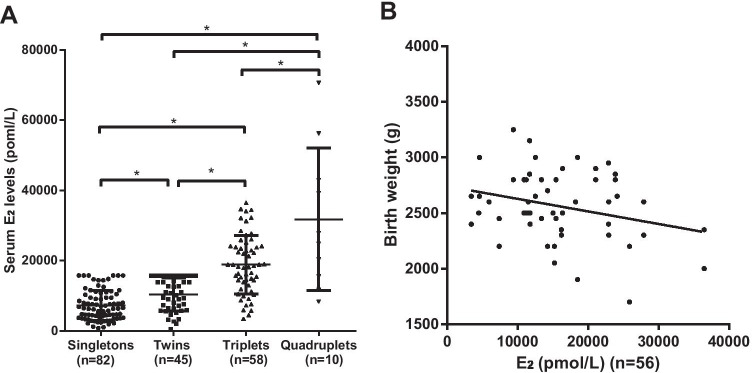
(**A**) Maternal serum E_2_ levels were significantly elevated at 8 weeks of gestation with an increasing number of fetuses. E_2_ levels were determined by electrochemiluminescence. E_2_ levels in serum samples from women with singleton pregnancy (*n* = 82), twin pregnancy (*n* = 45), triplet pregnancy (*n* = 58), or quadruplet pregnancy (*n* = 10) were compared by one-way ANOVA (*P < 0.05). (**B**) Multivariate correlation analysis showed that maternal serum E_2_ levels were inversely correlated with the birth weight of offspring (*n* = 56, r = −0.32, * *P* < 0.05)

We analyzed maternal serum E_2_ levels at 8 weeks of gestation (*n* = 195), including 82 singleton conceptions, 45 twin conceptions, 58 triplet conceptions, and ten quadruplet conceptions. The results showed that maternal serum E_2_ levels were significantly elevated with an increasing number of fetuses. The mean serum E_2_ concentrations were 10,322.79 ± 701.32 pmol/L for twin conceptions, 18,883.01 ± 8321.94 pmol/L for triplet conceptions, and 31,794.10 ± 6413.26 pmol/L for quadruplet conceptions, which were all significantly higher than that of singleton pregnancies (7240.09 ± 465.48 pmol/L, *P* < 0.01). Furthermore, there were significant differences in E_2_ levels among the four groups (*P* < 0.01) (Fig. 1A).

### The Association between Maternal Serum E_2_ Levels of Multiple Pregnancies at 8 Weeks Gestation and Offspring Birth Weight

We analyzed the correlation between maternal serum E_2_ levels of multiple pregnancies at 8 weeks gestation and the birth weights of pregnancies reduced to twins. The analysis showed that maternal serum E_2_ levels at 8 weeks gestation were negatively correlated with offspring birth weight (*r* = −0.32, *P* < 0.05, Fig. 1B), suggesting that offspring birth weight could be inversely affected by increasing maternal serum E_2_ levels.

### CDKN1C and DNMT1 Expression in Embryo Tissue, Newborn UCBMNC, and Placenta

**Fig. 2:**
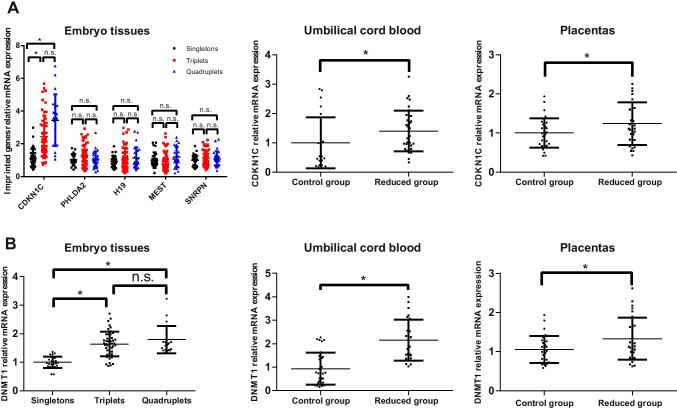
(**A**) Relative expression of imprinted genes in embryo tissues and *CDKN1C* in newborn UCBMNC and placenta tissues. (**B**) Relative *DNMT1* expression in embryo tissues, UCBMNC, and placenta tissues

qRT-PCR was performed on 77 early embryo tissues from selective MFPR and 29 embryo tissues from naturally conceived singleton pregnancies to investigate the mRNA levels of imprinted genes related to growth and development in multiple and singleton embryo tissue. The results showed that *CDKN1C* mRNA levels were significantly higher in early embryo tissue from triplet and quadruplet pregnancies than those in singleton pregnancies. The *CDKN1C* mRNA levels in embryo tissue from quadruplet pregnancies were also higher than those in triplet pregnancy embryo tissue; however, the difference was not significant. We further analyzed *CDKN1C* expression levels in newborn UCBMNC and placenta of 20 multiple pregnancies reduced to twins and matched primary twin pregnancies. Consistent with the results from the embryo tissue, *CDKN1C* expression levels in the UCBMNC and placenta from multiple pregnancies reduced to twins were significantly increased compared to those from the matched twin pregnancies (Fig. 2A). Significantly higher *DNMT1* expression levels were also observed in MFPR embryo tissue, UCBMNC, and placenta from multiple pregnancies reduced to twins compared to the controls (Fig. 2B).

### E_2_ Modulates the Expression of DNMT1 and CDKN1C mRNA Levels

**Fig. 3:**
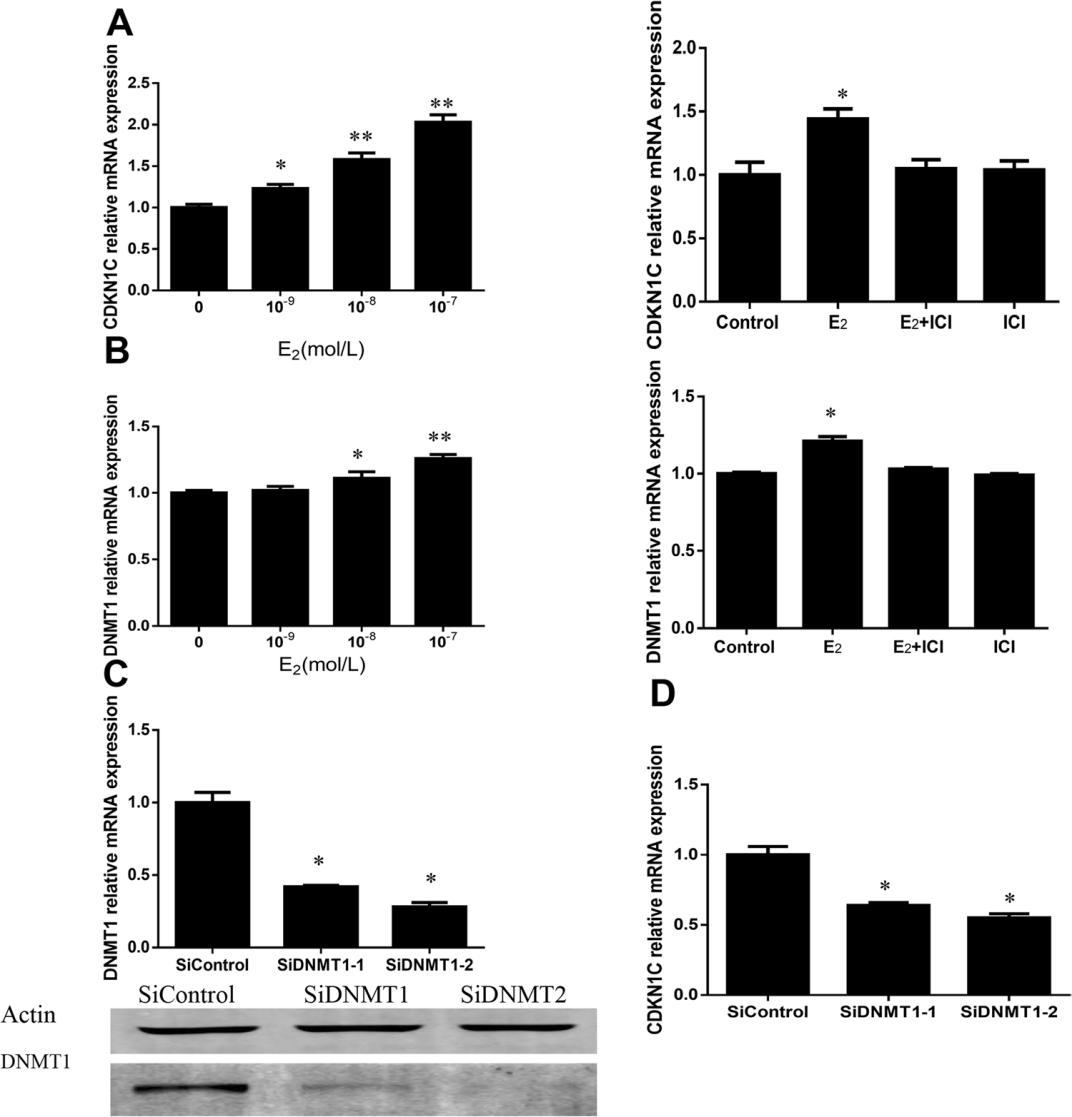
E_2_ increased *CDKN1C* and *DNMT1* expression in a dose-dependent manner. These effects were attenuated by ICI, an ER antagonist. (**A**) *CDKN1C* expression in HTR-8 cells after treatment with different doses of E_2_ for 24 h. The increased *CDN1C* mRNA expression levels induced by E_2_ were blocked by the estrogen blocker ICI182780 (10^–7^ mol/L) or co-treatment with E_2_ and ER antagonist ICI (10^–7^ mol/L) (*n* = 9). (**B**) *DNMT1* expression in HTR-8 cells after treatment with different doses of E_2_ for 24 h. The increased *DNMT1* mRNA expression levels induced by E_2_ were blocked by ICI (10^–7^ mol/L) (*n* = 9). Effects of *DNMT1* silencing on the expression of *DNMT1* and response to E_2_ induction in HTR8 cells, C and D. (**C**) HTR8 cells were transiently transfected with *DNMT1* or scrambled control siRNA. Forty-eight hours after transfection, cell samples were analyzed by qRT-PCR and western blotting. (**D**) HTR8 cells were transiently transfected with siRNA against *DNMT1* and then incubated with 10^−7^ M E_2_. *CDKN1C* expression was analyzed by qRT-PCR. DNMT1 knockdown decreased the responsiveness of HTR8 cells to E_2_ stimulation

We found that serum E_2_ levels were significantly higher in multiple pregnancies than those in singleton pregnancies and were elevated with the increasing numbers of fetuses. To investigate whether E_2_ could regulate *CDKN1C* and *DNMT1* mRNA levels, HTR-8 cells were treated with E_2_ and the ER inhibitor ICI182780. Treatment of estrogen-starved HTR8 cells with E_2_ for 24 h increased *CDKN1C* and *DNMT1* mRNA levels in a dose-dependent manner. However, incubation with ICI182780 significantly reduced the effects of E_2_ on *CDKN1C* and *DNMT1* mRNA expression (Fig. 3A and B). We next investigated the effect of *DNMT1* on *CDKN1C* expression. As shown in Fig. 3C, *DNMT1* siRNA effectively knocked down *DNMT1* expression. Notably, *DNMT1* knockdown reversed the E_2_-induced increase in *CDKN1C* mRNA levels, indicating that *DNMT1* mediates E_2_-induced upregulation of *CDKN1C* (Fig. 3D).

### KvDMR1 Hypermethylation Mediates E_2_-Induced CDKN1C Upregulation

**Fig. 4:**
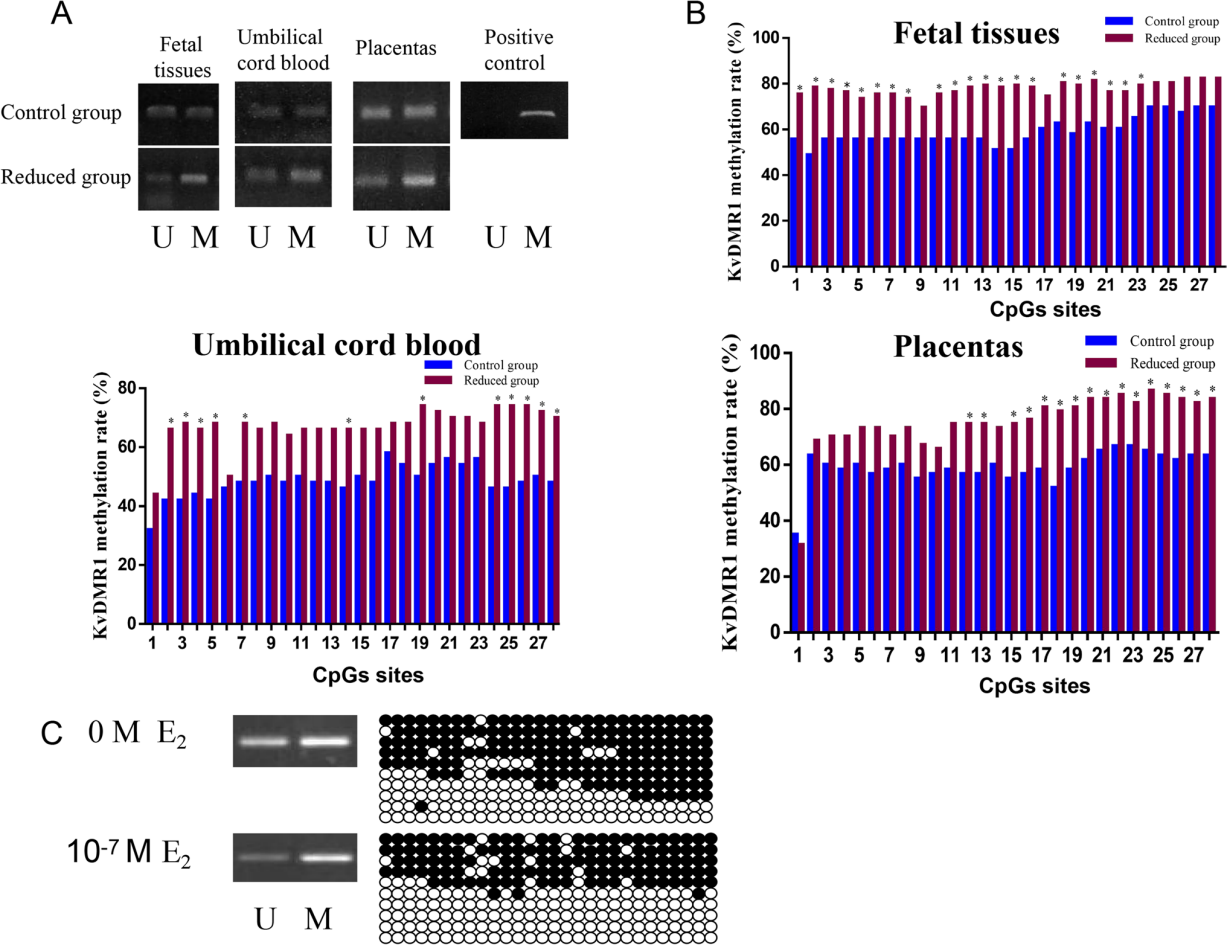
(**A**) Representative MSP results of DNA methylation. KvDMR1 was hypermethylated in MFPR embryo tissue, UCBMNC, and placenta of multiple pregnancies reduced to twins. (**B**) *KvDMR1* CpGs had significantly higher hypermethylation in embryo tissues of multiple pregnancies, UCBMNC, and placenta of multiple pregnancies reduced to twins, with 75.00% (21/28), 42.86% (12/28), and 57.14% (16/28) of the CpGs showing abnormal hypermethylation, respectively. (**C**) MSP and bisulfite sequencing analysis of *KvDMR1* methylation in HTR8 cells without or with 10^–7^ M E_2_ treatment. Ten independent clones of the indicated groups were sequenced. Black circles denote methylated cytosine in specific CpGs, and white circles denote nonmethylated cytosine in specific CpGs. U and M denote unmethylated DNA-specific and methylated DNA-specific amplification, respectively. P denotes the positive control

We examined the methylation status of *KvDMR1* in embryo tissue, UCBMNC, and placenta using MSP. We found that *KvDMR1* was hypermethylated in MFPR embryo tissues, UCBMNC, and placenta for multiple pregnancies reduced to twins (Fig. 4A). These results were further confirmed using bisulfite DNA sequencing (Fig. 4B). Furthermore, treatment of HTR8 cells with E_2_ led to *KvDMR1* hypermethylation (Fig. 4C). Together, these results suggested that *CDKN1C* upregulation was associated with the increased methylation of *KvDMR1*.

### DNMT1 Promoter Responses to E_2_ and Its Receptors

**Fig. 5:**
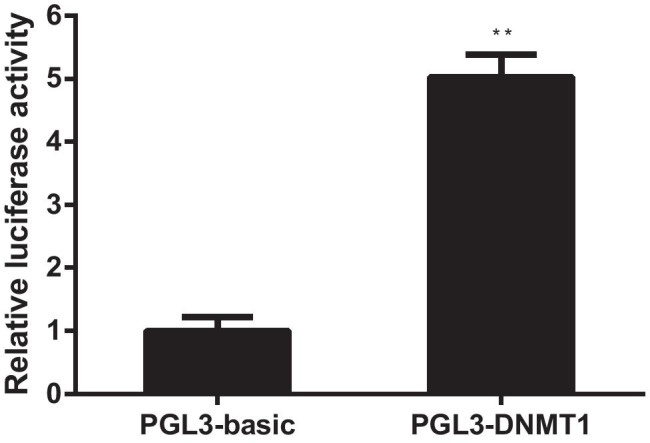
(**A**) E_2_ induced human *DNMT1* transcription through an ERE proximal to the transcription start site. HTR8 cells cultured in phenol red-free medium supplemented with 5% double-charcoal stripped FCS and transiently transfected with empty pGL3-basic or pGL3-DNMT1 (1 μg) with the treatment of E_2_. Cells were harvested 24 h after transfection and assayed for luciferase activity. DNMT1 promoter-mediated luciferase values were normalized to control *Renilla* luciferase values. (**B**) ERα binds directly to the ERE on the *DNMT1* promoter. ChIP analysis of the human *DNMT1* promoter was performed after HTR8 cells were treated with E_2_ for 24 h. ChIP assays were performed using IgG, anti-RNA polymerase, and anti-ERα antibodies as indicated. After cross-link reversal, the co-immunoprecipitated DNA was amplified by PCR using primers specific for the *DNMT1* ERE containing region (− 659 to − 647) or a control region (− 2123 to − 2111 or − 728 to − 716). The PCR products were resolved using a 2% agarose gel. Representative data from three independent experiments are presented

E_2_ is thought to exert its biological effects by binding to ERs [34]. To investigate if the regulation of *DNMT1* expression by E_2_ is at the promoter level, we determined the effect of E_2_ stimulation on the activation of a luciferase reporter driven by the proximal 2.4-kb fragment of the *DNMT1* promoter. As shown in Fig. 5, E_2_ induced a *DNMT1*-luc activity fivefold compared to the control. Three putative estrogen response elements (EREs) were located in the *DNMT1* promoter using the transcription factor binding sites and cis-regulatory search system (http://rsat.ulb.ac.be/). Furthermore, CHIP demonstrated that ERα could bind to the *DNMT1* promoter in HTR8 cells after treatment with E_2_ (Fig. 5). In particular, ERα bound to the ERE-like site at −659 bp of the *DNMT1* promoter. Collectively, these findings indicate that *DNMT1* was the downstream target of E_2_.

## Discussion

Many studies have shown that MFPR is associated with a reduced risk of preterm birth, LBW, and no increase in pregnancy loss compared to that in unreduced multiple pregnancies [28, 35–37]. The perinatal outcomes of twin pregnancies after early and late MFPR appear similar [38]. MFPR has been widely accepted as a legitimate treatment option for patients with triplets or higher-order pregnancies [36]. However, these previous studies all compared obstetric outcomes before and after MFPR. To date, only three studies have compared reduced and unreduced multiple pregnancies. Mheen et al. [39] reported that the median neonatal birth weight in multiple pregnancies reduced to singleton pregnancies was significantly lower than that in primary singleton pregnancies. Razaz et al. [37] found that the rate of very low birth weight was significantly higher among pregnancies reduced to twins than unreduced twin pregnancies. Zipori et al. [40] found that the incidence of SGA was not different between the MFPR and unreduced triplet groups. Our findings are consistent with these results. In the current study, we found an increased risk of LBW and SGA when maternal serum E_2_ levels were high in the first trimester of multiple pregnancies. Although the results for pregnancies reduced to singletons were better than those reduced to twins, the mean birth weight of pregnancies reduced to singletons was still significantly lower than that of primary singletons. When we excluded the effects of the duration of gestation, there was a higher incidence rate of SGA in multiple pregnancies reduced to singletons and twins. These results indicate that some effects of the first trimester of multiple pregnancies persist and cannot be completely eliminated by fetal reduction.

Maternal serum E_2_ levels in the first trimester of pregnancy were increased with the number of fetuses and negatively correlated with offspring birth weight. E_2_ is the most bioactive estrogen. In mice, high E2 exposure during early pregnancy decreases offspring body weight [41]. Moreover, high E_2_ doses cause embryonic mortality and fetal and placental growth retardation in rats [42]. However, the mechanisms by which the dramatic increase in E_2_ levels in pregnant women adversely affects fetal development and growth during the first trimester of multiple pregnancies are unknown.

Evidence from animals and humans has indicated that certain transient environmental influences can produce persistent changes in epigenetic marks that last throughout life [14]. Imprinted genes are implicated in the regulation of fetal growth and development and are especially sensitive to environment-mediated changes in DNA methylation [43]. Endocrine disruptors can alter DNA methylation patterns of key genes, resulting in transcriptional changes [44]. Cases of ART-conceived children afflicted with imprinting mutations have been reported [45]. *CDKN1C* is a maternally expressed imprinted gene with a major role in regulating embryonic growth. Previous studies demonstrated that excess CDKN1C could cause embryonic growth retardation in animals and humans [46, 47]. In the present study, we found that the expression levels of *CDKN1C* and *DNTM1* were significantly upregulated in embryo tissue from multiple pregnancies compared to singleton pregnancies.

The expression of imprinted genes is regulated by the methylation status of the CpGs in the differentially methylated regions (DMRs) of each gene [48]. DNMTs are responsible for DNA methylation, and high *DNMT1* activity contributes to abnormal hypermethylation of certain genes [15]. These observations suggest that E_2_ might interact with *DNMT1* and positively regulate its enzymatic activity. *DNMT1* overexpression leads to hypermethylation of *KvDMR1*. *KvDMR1* is the predominant imprinting control region of *CDKN1C* on human chromosome 11p15. We found that the methylation levels of *KvDMR1* and the DMR of *CDKN1C* are significantly increased in response to E_2_, resulting in the *CDKN1C* upregulation. This upregulation could contribute to the LBW and SGA of multiple pregnancies. Moreover, increased *CDKN1C* and *DNMT1* expression levels were also associated with the hypermethylation of *KvDMR1* in UCBMNC and placenta of multiple pregnancies reduced to twin pairs, indicating that transiently high serum E_2_ levels might induce persistent changes in epigenetic marks during early gestation.

To confirm that *CDKN1C* expression was regulated by high E_2_ levels, we further investigated the regulation of *CDKN1C* expression by E_2_. In the “classical” mechanism of estrogen action, the long-term effects of E_2_ are generally ascribed to transcriptional modulation of target genes through the binding of estrogen receptors (ERs). These receptors modulate gene expression directly through EREs in the promoter regions of target genes or indirectly through interaction with transcription factors, co-activators, or transcription complexes [49, 50]. Using a reporter gene assay, we demonstrated that ERα activated *DNMT1* transcription through an ERE located at −659 bp upstream of the transcriptional starting site. The palindromic sequence of the ERE in the *DNMT1* promoter was similar to the canonical ERE [51]. The direct binding of ERα to the *DNMT1* promoter was confirmed by ChIP. Collectively, our results clearly confirmed that *CDKN1C* acted as a key mediator of E_2_ effects in HTR-8 cells.

To the best of our knowledge, this study is the first to demonstrate that high E_2_ levels in the first trimester of multiple pregnancies are an independent risk factor for SGA. This conclusion was based on a single-center study with a relatively large sample size. However, we acknowledge that the study has some limitations. First, it was retrospective in design, and E_2_ levels post-reduction could be evaluated in a prospective study. Second, multiple pregnancies were achieved through ART, which included controlled ovarian hyperstimulation and IVF. Therefore, it is hard to exclude the effects of infertility on our findings. Third, pregnancy reduction from twin to singleton is not as commonly accepted as reducing higher-order multiples. Thus, the *DNMT1* and *CDKN1C* expression levels in singletons from reduced twins could not be obtained.

## Conclusions

The present study demonstrated that high maternal E_2_ levels might induce high *CDKN*1C expression, leading to LBW and SGA in the first trimester of multiple pregnancies. To minimize the risks of LBW and SGA, abnormally high maternal serum E_2_ levels during early pregnancy in ART patients should be avoided.

## Supplementary Information

Below is the link to the electronic supplementary material.

Supplementary file1 (DOC 29 KB)

## Data Availability

All raw data and material are available upon request.
