# Supplementary material for: High Maternal Serum Estradiol in First Trimester of Multiple Pregnancy Contributes to Small for Gestational Age via DNMT1-Mediated CDKN1C Upregulation
Source: Reprod Sci. 2021 Sep 27;29(4):1368–78. doi: 10.1007/s43032-021-00735-8 (PMC8907102; doi:10.1007/s43032-021-00735-8)
Supplement: Supplementary file 1 — Supplementary file1 (DOC 29 KB) [file 43032_2021_735_MOESM1_ESM.doc]

Supplemental Table 1. Primers used for real-time quantitative RT-PCR (qRT-PCR).

| Gene | Sense 5’-3’ | Antisense 5’-3’ | Size(bp) |
| --- | --- | --- | --- |
| CDKN1C | CTGATCTCCGATTTCTTCGC | TCTTTGGGCTCTAAATTGG | 161 |
| DNMT1 | TGGACGACCCTGACCTCAAAT | GCTTACAGTACACACTGAAGCA | 168 |
| GAPDH | CAGGGCTGCTTTTAACTCTGG | TGGGTGGAATCATATTGGAACA | 102 |

Supplemental Table 2. Primers used for methylation speciﬁc PCR (MSP).

| Primer set | Sense 5’-3’ | Antisense 5’-3’ | Size(bp) |
| --- | --- | --- | --- |
| KvDMR1M | TTTTTTCGGTTAATGATAGGATACG | TCTACCTAAAAACTACGACAACGCT | 178 |
| KvDMR1U | TTTTTTGGTTAATGATAGGATATGG | TCTACCTAAAAACTACAACAACACT | 171 |

The M and U recognize the methylated and unmethylated sequences, respectively. The size of the PCR product is indicated in each case.

Supplemental Table 3. Primers used for bisulfite sequencing (BSP).

| Primer set | Sense 5’-3’ | Antisense 5’-3’ | Size(bp) |
| --- | --- | --- | --- |
| KvDMR1 outer | GTTTTTTGTTAGGTGGGTGGTTTG | TTACTAAAAAACTCCCTAAAAATCC |  |
| KvDMR1 inner | GTGGTGAATATATTAYGTAGAGAATTGGTT | TTCTACCTAAAAACTACRACAACGCTCCGA | 387 |

Y: C or T; R: G or A

Supplemental Table 4. DNMT1 primers used for PCR.

| Primer set | Sense 5’-3’ | Antisense 5’-3’ | Size(bp) |
| --- | --- | --- | --- |
| DNMT1  (-659/-647) | AGGCTGGAATGTAGTGGTA | TAGTCGTTGTGGCATGTG | 119 |
| DNMT1  (-2123/-2111) | GCAATCTCGGCTCACTG | AACCCTGTCTCTCCTCAAA | 132 |
| DNMT1  (-728/-716) | CTAACCTCAAGCGATCCT | GAGACAGAGCAAGACCTT | 140 |
